# Thermodynamics of Surfactant-Enriched Binary-Fluid Systems

**DOI:** 10.1021/acs.langmuir.4c01724

**Published:** 2025-01-21

**Authors:** Tom B. van Sluijs, Stein K. F. Stoter, E. Harald van Brummelen

**Affiliations:** Eindhoven University of Technology, P.O. Box 513, 5600 MB Eindhoven, The Netherlands

## Abstract

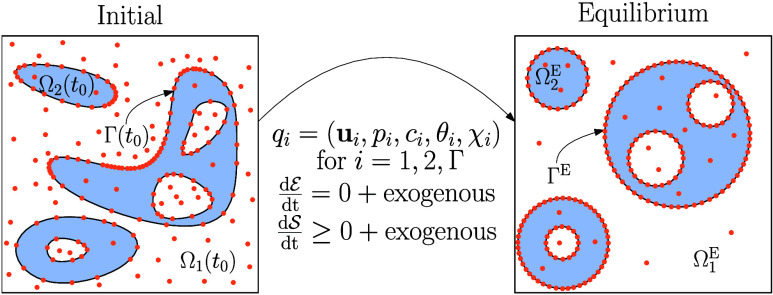

Surface-active agents (surfactants) release potential energy as they migrate from one of two adjacent fluids onto their fluid–fluid interface, a process that profoundly impacts the system’s energy and entropy householding. The continuum thermodynamics underlying such a surfactant-enriched binary-fluid system has not yet been explored comprehensively. In this article, we present a mathematical description of such a system, in terms of balance laws, equations of state, and permissible constitutive relations and interface conditions, that satisfies the first and second law of thermodynamics. The interface conditions and permissible constitutive relations are revealed through a Coleman–Noll analysis. We characterize the system’s equilibrium by defining equilibrium equivalences and study an example system. With our work, we aim to provide a systematically derived framework that combines and links various elements of existing literature, and that can serve as a thermodynamically consistent foundation for the (numerical) modeling of full surfactant-enriched binary-fluid systems.

## Introduction

Surface active agents (surfactants) are amphiphilic molecules, meaning that they comprise both hydrophobic and hydrophilic parts, that minimize potential energy by migrating to fluid–fluid interfaces to locate these hydrophobic and hydrophilic parts in the respective phases. On interfaces, these molecules reduce the surface tension due to a reduction of the interfacial energy density by disrupting molecular bonds.^1,2^ They are of key importance in numerous and widely varied settings, ranging from the coffee-stain effect^3−5^ and household detergents^6^ to industrial applications such as emulsifiers in the food industry,^7−15^ inkjet printing^4,12−17^ and biomedical applications.^18−24^

Given the wide range of applications, understanding the complex influence of surfactants on a binary fluid system, i.e., a system composed of two immiscible fluid components separated by an interface, is crucial. Models are indispensable for understanding and predicting the impact of surfactants on dynamical processes and, consequently, many types of models exist. The dynamics of interfaces have been explored for material and immaterial interfaces,^25^ in conjunction with inviscid as well as viscous (e.g., Boussinesq-Scriven) interface stress responses.^26^ The surfactant transport from, to, and across the interface can be modeled based on statistical mechanics considerations,.^27^ Various so-called “isotherms” have been formulated that describe the bulk-to-interface distribution of surfactant concentration,^27^ for example Henry isotherm, Langmuir isotherm, Freundlich isotherm, Volmer isotherm, and Frumkin isotherm.^27−29^ Implicitly, these isotherms also define the surfactant-concentration influence on the surface tension,^27,28^ which dictates the Young–Laplace pressure across the interface, as well as the Marangoni flow along the interface.^16^

In order to (computationally) describe a complete binary-fluid surfactant system, these component-level models must be combined. Notable efforts are those by Lebon, Jou and Casas-Vázques who describe nonequilibrium thermodynamics of coupled problems,^30^ Venerus and Öttinger who describe the general thermodynamics of multiphase systems with interfaces (but without surfactants),^31^ Zhang et al., who derive a continuum model with insoluble surfactants on the interface,^32^ and works by Garcke, Thiele, Zhao and co-workers that introduce soluble surfactants.^29,33,34^ Such system-level models enable numerical study of complex cases, as overviewed in.^4^ Examples range from the investigation of the influence of surfactants on topological changes^35,36^ to studies on gravitational effects in surfactant-enriched multiphase flows^37,38^ as well as the effect of surfactants on evaporation.^39,40^

These continuum models are based on systems of partial differential equations equipped with suitable auxiliary conditions; state variables characterize the state of physical systems and the dynamic evolution of these states is governed by balance-laws. The balance laws involve auxiliary variables that drive the evolution, for which closure relations follow from constitutive relations that describe the material response to the system’s state based on physical parameters. The laws of thermodynamics dictate which system behavior is permitted. Coleman and Noll introduce the application of a thermodynamic framework to a classic continuum-mechanics system.^41^ In this setting, the first and second law of thermodynamics, describing the balance of total energy and entropy, respectively, lead to general conditions for admissible constitutive relations.

Despite the importance of binary-fluid-surfactant systems and the wide range of existing work, the available literature lacks a complete and coherently derived continuum-thermodynamics-based model. This article contains the first presentation of such a systematic derivation, where we methodically retrieve and extend on elements of the theory as presented by Lebon, Jou and Casas-Vázques,^30^ Venerus and Öttinger,^31^ and Garcke, Lam, and Stinner,^29^ and found in other earlier cited works. Specifically, we consider a general binary-fluid-surfactant system, under the (standard) assumption that the mass carried by the interface is negligible. We use a procedure and argumentation analogous to Coleman and Noll^41^ to derive thermodynamically imposed constraints on constitutive relations, equations of state, and interface-bulk coupling conditions. In particular, our derivation naturally yields an equation of state for the interface pressure, distinctly highlighting energetic and entropic contributions. Similarly, we obtain the Young–Laplace pressure jump over an interface, and the thermal and concentration-based Marangoni stresses from the interface momentum balance. Additionally, we acquire an expression for surfactant adsorption and an equilibrium surfactant concentration distribution between the bulk fluids and the interface. This expression balances the thermal entropy increase via the release of mixture energy and the effect of configurational entropy. Finally, we present a new and rigorous characterization of equilibrium of the binary-fluid-surfactant system based on equilibrium equivalences. The general theory is illustrated by giving an example of equilibrium states as a function of temperature and bulk surfactant concentration.

The presented thermodynamic framework provides a fundamental theory for constructing models for interfacial and bulk properties and interface-bulk-interaction conditions, in the form of equations of state, constitutive relations, and interface conditions, in such a manner that the resulting models comply with the first and second laws of thermodynamics, and exhibit a meaningful equilibrium. The framework incorporates nonequilibrium effects, in that the equilibrium interface conditions, e.g., continuity of temperature, are not imposed but, instead, these emerge as consequences of the second law in combination with the equilibrium equivalences.

The remainder of this article is structured as follows: in System Description section we describe the physical system in terms of the relevant state variables and introduce the modeling paradigm. The system’s evolution equations are derived in Conservation Laws section, based on first-principles, and in Constitutive Relations and Equations of State section we perform a Coleman-Noll analysis to derive the permissible constitutive relations, interface conditions, and necessary equations of state. In Equilibrium section we consider the equilibrium equivalences of the system and show an equilibrium example. Finally, we draw conclusions in Conclusions section.

## System Description

This section presents the considered binary-fluid-surfactant system and modeling paradigm. The binary-fluid system is set on an ambient domain, and we derive the balance laws for an arbitrary control volume in this ambient domain. In addition, we introduce the state variables, auxiliary variables, and state functions, which are used in the balance laws. The modeling paradigm consists of postulates and modeling assumptions.

### Setting and Notation

**Figure 1:**
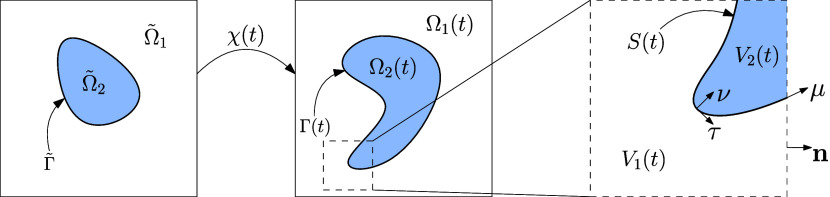
Schematic depiction of the modeled system, illustrated by the reference domain, domain, and control volume, from left to right, respectively. Ω̃_*i*_ for i = 1, 2 and Γ̃ represent the reference bulk domains and the reference interface, respectively, Ω_*i*_(*t*) for *i* = 1, 2 and Γ(*t*) are the time-dependent counterparts after the application of the bijective deformation map **χ**(*t*). The control volume *V*(*t*) contains the subvolumes *V*_*i*_(*t*) for i = 1,2 and the control surface *S*(*t*). On the control surface, we denote by **ν** the unit normal vector on the interface, directed external to Ω_1_(*t*), and by **τ** the tangent plane to Γ. ***n*** is the outer unit normal vector of the control volume and **μ** is the unit conormal vector on the intersection of the interface with the control-volume boundary.

We consider a binary-fluid-surfactant system composed of two immiscible fluid species separated by an interface, with a surfactant that is soluble in both fluid species. To provide a setting for the binary-fluid-surfactant system, we regard a time interval  and a spatial domain  (*d* = 2, 3). The two fluid species reside in two complementary bulk domains Ω_1_(*t*) and Ω_2_(*t*), and are separated by an interface Γ(*t*) = ∂Ω_1_ ∩ ∂Ω_2_ of codimension 1; see Figure 1 for an illustration. We assume that the time-dependent domains Ω_*i*_(*t*) are images of initial reference configurations Ω̃_*i*_ under time-dependent deformation maps **χ**_*i*_(*t*, ·): Ω̃_*i*_ → Ω corresponding to the flow of the binary fluid. That is, Ω_*i*_(*t*) = **χ**_*i*_(*t*; Ω̃_*i*_) and **χ**_*i*_(0, ) =  for all  ∈ Ω̃_*i*_ , and  = ∂_*t*_ **χ**_*i*_ corresponds to the velocity of fluid component *i*, expressed in the reference configuration. For convenience, we also define the compounded map **χ**(*t*, ·): Ω̃_1_ ∪ Ω̃_2_ → Ω such that the restriction  coincides with **χ**_*i*_, and extend this notational convention to other quantities defined on Ω_1_ ∪ Ω_2_. The binary-fluid velocity in the actual time-dependent configuration is then ***u***(*t*, ·) = ***ũ***(*t*, **χ** ^–1^(*t*, ·)). As we will allow the binary-fluid components to slip along the interface, **χ** is not generally continuous across the interface in its tangential component. We assume that the subdomain maps **χ**_*i*_ and their inverses are continuous, and that **χ**_1_(·, Γ̃) = **χ**_2_(·, Γ̃) (in the sense of traces), i.e., Ω_1_ and Ω_2_ remain connected. The continuity conditions imply that we will not regard topological changes of the interface. The connectedness condition implies that the binary fluid velocity is continuous across the interface in its normal direction, i.e., ***u***_1_· **ν** = ***u***_2_ · **ν**, with **ν** the exterior unit normal vector of Ω_1_(*t*) on the interface.

To derive the governing equations for the binary-fluid-surfactant system, we consider a control volume *V*(t) ⊆ Ω that contains the bulk fluids in the subvolumes *V*_*i*_(*t*) = Ω_*i*_(t) ∩ *V*(*t*) and the interface in the control surface *S*(*t*) = Γ(t) ∩ *V*(*t*). For any point on the interface, we denote by **τ** the tangent plane to Γ. For any point on the intersection of the interface with the control-volume boundary, Γ(t) ∩ ∂*V*(*t*), we denote by **μ** the unit conormal vector; see Figure 1. The external unit vector to ∂*V*(*t*) is indicated by ***n***.

In the model that we consider, the state of the binary-fluid-surfactant system is characterized by the state *y* which contains the fields of state variables, as shown in Table 1.

**Table 1:** Variables, with *i* ∈ {1, 2, Γ} for Bulk 1, Bulk 2 and Interface Quantities

**State variables**	**Symbol**
Velocity	***u***_*i*_
Pressure	*p*_*i*_
Surfactant concentration	*c*_*i*_
Thermal energy density	*e*_th,*i*_
Domain map	**χ**_*i*_
	
**Auxiliary variables**	**Symbol**
Deviatoric stress	**τ**_*i*_
Concentration flux	***J***_*i*_
Heat flux	***q***_*i*_
Heat source	Σ_*i*_
	
**State functions**	**Symbol**
Mixture energy density	*e*_m,i_(*c*_*i*_)
Entropy density	*s*_*i*_(*e*_th,*i*_ , *c*_*i*_)

### Postulates and Model Assumptions

We model the binary-fluid-surfactant system in the framework of continuum thermodynamics. The general postulates of this framework comprise conservation laws and the laws of thermodynamics. Specifically, the balance of mass, momentum, concentration, and thermal energy. The first law of thermodynamics pertains to conservation of energy. We consider three contributions to the total energy: kinetic energy, thermal energy, and mixture energy. The second law of thermodynamics dictates non-negative entropy production. Within this framework, irreversible processes produce entropy and reversible processes do not.

Additionally, we define the following model assumptions which will be used throughout the remainder of this article:

MA-1: The bulk phases have constant mass density.
MA-2: The interface neither carries nor transfers mass.
MA-3: There are no exogenous sources to any of the balance laws.

Assumption MA-1 implies that the presence of surfactants does not significantly affect the mass density in the bulk domains. This assumption is in agreement with the fact that in most practical applications of surfactants, the surfactants are added in relatively low concentrations and, hence, their overall contribution to mass density is small. Assumption MA2 is a standard assumption in most models of binary-fluid flows. Because the thickness of a binary-fluid interface is generally only a few Å,^42,43^ the mass density with respect to surface measure is negligible. For instance, the kinetic energy of the interface is orders of magnitude smaller than its surface tension. Conversely, if surface mass density is not disregarded, then the equation of motion for the interface must be retained in the model, which yields a profound complication; see, for instance, ref (44). The second negation in MA2 means that there is no transfer of mass between the bulk phases. It is to be noted that MA2 does not preclude the transport of surfactants from the bulk phases to the interface and vice versa. Even if surfactant is present at the interface in large concentrations (with respect to surface measure), the surface mass density is generally negligible nonetheless. Assumption MA3 is nonessential, and merely serves to facilitate the presentation.

The behavior of the binary-fluid-surfactant system is to a large extent determined by properties of the state functions in Table 1. The state functions are point-wise functionals of the state variables. We insist that the state functions satisfy the following state-function assumptions:

SA-1: The mixture energy densities  are twice continuously differentiable and strictly convex.
SA-2: The entropy density  is twice continuously differentiable and strictly concave with respect to both arguments, and is strictly increasing with respect to its first argument (viz. the thermal energy density).

To further elucidate the model, the arguments of *e*_m,i_ and *s*_*i*_ have been indicated in Table 1.

## Conservation Laws

A full description governing the evolution of variables in Table 1 will require balance laws, constitutive relations, and equations of state. In this section, we focus on the balance laws following from the conservation of mass, momentum, and surfactant concentration, as well as the conservation of thermal energy and total energy, i.e., the first law of thermodynamics.

### Balance Laws in Bulk Phases

The transport of mass, momentum, surfactant concentration, and thermal energy can, at the continuum level, be described by conservation laws that involve fluxes, sources, and sinks. The partial differential equations then follow from balance equations at arbitrarily chosen control volumes. Despite the textbook nature of this material, we present it here to facilitate its adaptation on submanifolds, i.e., the interface, later on.

Consider an arbitrary extensive quantity *C*, the total of which is defined on a material control volume *V*(*t*), i.e., one that moves with velocity ***u***, as the integral of its respective density *c*
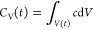
1The time-rate of change of *C*_V_ depends on the flux ***J*** through the boundary of the control volume and on the source density *r* in the control volume
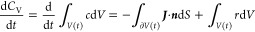
2Applying Reynolds’ transport theorem and the divergence theorem yields
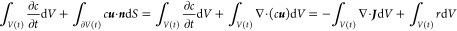
3As this equality holds for arbitrary choice of *V*(*t*), the integrands must coincide
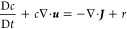
4with  the total derivative.

Specifying the general balance law (4) to the balance of mass, with density ρ_*i*_ , for which there is no driving flux and there are no sources, we obtain
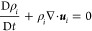
5From assumptions MA1 and MA3 in Postulates and Model Assumptions section, it follows that the material derivative of the mass density is zero. Equation 5 reduces to the condition that the velocity field is solenoidal
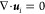
6Solenoidality of ***u*** implies that the second term in the left member of the general balance equation (4) vanishes. The balance of linear momentum, corresponding to density ρ_*i*_***u***_*i*_ and with the (negative of the) Cauchy stress **σ** = **τ**_*i*_ – *p*_*i*_***I*** as driving flux, the surfactant concentration corresponding to density *c*_*i*_ and flux ***J***_*i*_, and the thermal energy with density *e*_th,*i*_ , flux ***q***_*i*_ and internal source Σ_*i*_ , can be condensed into, respectively
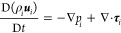
7
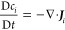
8
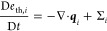
9

### Interface Balance Laws

Next, we consider a material control surface *S*(t) ⊂ Γ(*t*) that resides on the interface, i.e., a submanifold moving through the higher-dimensional volumetric domain. The generic balance equation for such a control surface reads
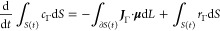
10where *c*_Γ_ and *r*_Γ_ represent surface density and surface source density, respectively. The vector **μ** is the in-plane outward facing normal to the boundary of the surface control volume; see Figure 1. The flux ***J***_Γ_ is tangential to the interface.

In this setting, Reynolds’ transport theorem and the divergence theorem take on the following forms
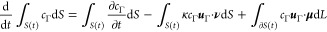
11
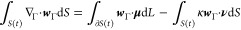
12where the vector **ν** is the out-of-plane normal to *S*(*t*) external to Ω_1_, ∇_Γ_ = ***I***_Γ_ · ∇ = (***I*** – **ν**⊗**ν**) · ∇ is the surface gradient, and κ = −∇_Γ_ · **ν** the total (additive) curvature,^45^ i.e., *d* – 1 times the mean curvature. Any vector ***w***_Γ_ on the interface can be decomposed into its in-plane component ***w***_Γ,**τ**_ = **P**_**τ**_***w***_Γ_ ≔ **ν** × ***w***_Γ_ × **ν** and out-of-plane component *w*_Γ, ***ν***_***ν*** = (**ν**·***w***_Γ_)**ν** according to ***w***_*i*_ = ***w***_Γ,**τ**_ + *w*_Γ, **ν**_***ν***. The surface divergence term in (12) can thus be recast into
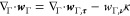
13

We tacitly extend quantities in bulk domains to the boundaries in the trace sense, e.g.,  and, accordingly, extend the above decomposition and operators to bulk vectors via the restriction of their trace to Γ, e.g., ***u***_*i*,**τ**_ = .

We elaborate on the surface source density by combining bulk boundary terms and interface balance laws from (2), (3), and (10) in a control volume encompassing parts of all domains. The influx from the bulk fluids is separated from the general source term as *r*_Γ_ =  · **ν** + Σ_Γ_, where the jump operator  is defined as  = *f*_1_ – *f*_2_. The term  represents the transfer of *c*_*i*_ from the bulk domains to *c*_Γ_ on the interface as a result of the bulk flux ***J***_*i*_ and the interface material control surface moving through the volumetric domain with velocity ***u***_Γ_.

Making use of the three identities (11)–(13) and noting that the flux vector ***J***_Γ_ is in-plane, i.e., ***J***_Γ_ · **ν** = 0, Equation 10 is equivalent to
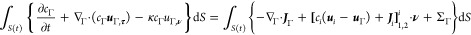
14As the choice of *S*(*t*) is arbitrary, the equality in (14) must in fact hold for the integrands, which leads to the general form of the balance law in partial-differential form
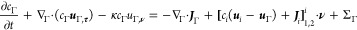
15

Specializing (15) to conservation of mass, we obtain the following differential equation
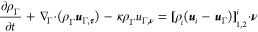
16

Assumption MA2 in Postulates and Model Assumptions section implies that the surface mass density in the left member of (16) vanishes according to ρ_Γ_ → + 0. From the second negation in MA2 it follows that ρ_*i*_(***u***_*i*_ – ***u***_Γ_)· **ν** = 0. Accordingly, the right member of (16) vanishes as well. Assumption MA2 is therefore consistent with conservation of mass on the interface. For later reference, we notice that the second negation in MA2 is equivalent to
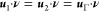
17

Let us note that the first identity is consistent with the condition on connectedness of the fluid-component subdomains stipulated in Setting and Notation section.

Just like for the bulk phases, the mass-balance identities simplify the general conservation equation (15). For the conservation of momentum, surfactant concentration, and thermal energy we then find
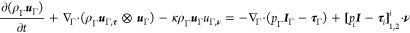
18
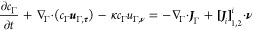
19
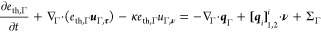
20Thermal energy is conventionally defined with respect to mass measure. However, because we assume that the interface does not carry mass, we use a thermal energy with respect to surface measure for completeness of the derivation.

Invoking again ρ_Γ_ → + 0 according to assumption MA2, the momentum balance (18) yields an instantaneous stress balance relation
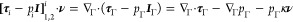
21Equation 21 conveys that the jump of the bulk surface tractions contains a contribution of the deviatoric interface stress tensor, a tangential Marangoni component and a normal Young–Laplace pressure jump, both related to the surface pressure. The surface pressure that has been introduced in the surface equation of motion (18) can therefore be identified with the negative surface tension, i.e., *p*_Γ_ = −σ_Γ_. It is important to note that assumption MA2 and the corresponding reductions of (16) to (17), and of (18) to (21), imply that equations of state are required for the interface velocity ***u***_Γ_ and surface pressure *p*_Γ_ as their evolution equations degenerate. This will be further considered in Constitutive Relations and Equations of State section.

### The First Law of Thermodynamics

The first step in deriving coupling relations between the previously derived evolution equations is to consider the first law of thermodynamics: the conservation of the total energy of the binary-fluid-surfactant system. The total system energy comprises three contributions, viz. kinetic energy, thermal energy and mixture energy
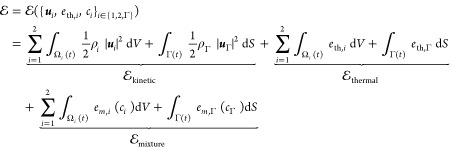
22where *e*_*m*,*i*_ (*c*_*i*_) and *e*_*m*,Γ_ (*c*_Γ_) are the bulk and interface mixture energy density, respectively. These energy densities are response functions based on the local surfactant concentrations and solely represent the energetic effect of the surfactant concentration. Let us note that the mixture energy also contains an interface-energy contribution. In view of its dependence on the interface surfactant concentration *c*_Γ_, we have classified this term as a mixture-energy term.

The first law of thermodynamics states that on an arbitrary material control volume *V*(*t*), the time rate of change of the total energy must equal the transport of energy into or out of the control volume. That is
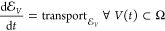
23where *V*(*t*) may intersect the interface, with *V*_1_(*t*) = *V*(t) ∩ Ω_1_(*t*), *V*_2_(*t*) = *V*(t) ∩ Ω_2_(*t*) and *S*(*t*) = *V*(t) ∩ Γ(*t*).

The definition of the right-hand-side transport term in (23) follows directly from the dependency of the energy functional on the state variables (definition (22)) and the equations governing the transport of these state variables (Equations 7 to 9 and 18 to 20)
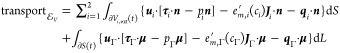
24with  as the derivative of the response function to its argument. Physically, these terms can be recognized as the mechanical work exerted on the boundary of the control domains, the in- or outflux of mixture energy, and the in- or outflux of thermal energy.

The left-hand-side term in (23) can independently be manipulated as follows. First, we make use of assumption MA2 to negate the contribution of the interface kinetic energy to the total energy. This leaves
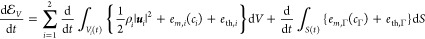
25We note that, despite the interface kinetic energy now equaling zero, it is important to formally include the dependency of the total energy on ***u***_Γ_ in definition (22): the variation of the total energy with respect to that argument gives rise to the nonvanishing mechanical work term on the interface boundary in Equation 24.

Upon invoking the bulk and interface Reynolds transport theorems, the bulk and interface divergence theorems, and recalling the solenoidality of the velocity field in the bulk phases, we obtain
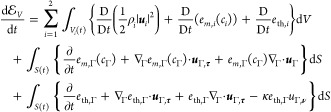
26

Appropriate expressions for the rates of change of the various quantities can be deduced from the earlier conservation laws. By multiplying the momentum conservation statement (7) by ***u***_*i*_, one finds an expression for the evolution of the kinetic energy
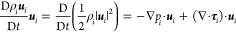
27where the first equality is due to the constant density assumption MA1. Applying the chain rule and making use of Equations 8 and 19, we obtain evolution equations for the bulk and interface mixture energy
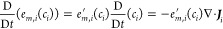
28
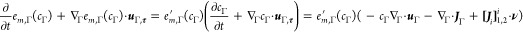
29Substituting the above equalities, as well as the balance laws for thermal energy, Equations 9 and 20, into Equation 26 results in
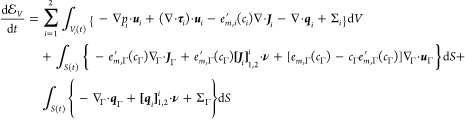
30

Next, we perform integration by parts on the bulk integrals. In particular, for the first and second term on the right-hand side of (30) we obtain
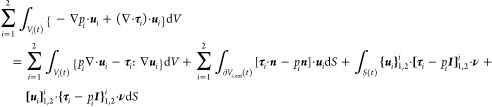
31where  denotes the average across the interface, *S*_Γ_ = *V* ∩ Γ and ∂*V*_*i*,ext_ = ∂*V*_*i*_ /*S*_Γ_. Note that  =  by virtue of the continuity of the normal component of the velocities across the interface according to Equation 17. In addition, ∇ · ***u***_*i*_ vanishes on account of (6). By adding the partition of zero ***u***_Γ_ – (***u***_Γ,**τ**_ + ***u***_**ν**_) to  in (31), it follows from the reduced interface-momentum balance (21) in conjunction with (30) that
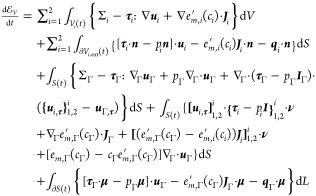
32where the second and last integrals now match the transport terms identified in Equation 24.

Substituting Equations 32 and 24 into 23, the first law of thermodynamics dictates
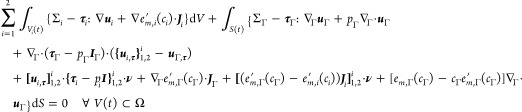
33As the control volume *V* is arbitrary, the integrands must vanish. It therefore holds that
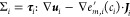
34
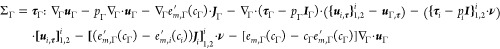
35

## Constitutive Relations and Equations of State

Constitutive relations for the fluxes must be such that the evolution of the system adheres to the second law of thermodynamics: any subsystem’s entropy is nondecreasing up to exogeneous influences. This section exposes the permissible forms of the constitutive relations through a Coleman-Nole procedure.

### The Second Law of Thermodynamics

The total entropy of the system is comprised of bulk and interface contributions
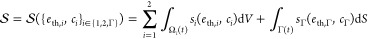
36with *s*_*i*_ = *s*_*i*_(*e*_th,*i*_ , *c*_*i*_) (*i* ∈ {1, 2, Γ}) the entropy density as a function of the thermal energy and the surfactant concentration.

The second law of thermodynamics insists that on an arbitrary material control volume *V*(*t*), the time rate of change of the total entropy cannot subceed the transport of entropy into or out of the control volume. That is
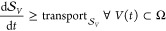
37

Analogous to the procedure performed to obtain the transport of total energy in (24), the definition of the right-hand-side transport term in (37) follows from the dependency of the entropy functional on the state variables (defined in (36)) and the equations governing the transport of these state variables (per Equations 7 to 9 and 18 to 20)
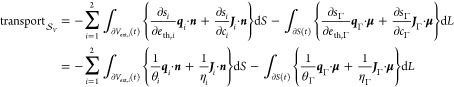
38where, like before, ∂*V*_ext,*i*_ = ∂*V* ∩ Ω_*i*_ , and ∂*S* is the boundary of *S*. Here,  (*i* = 1, 2, Γ) may be identified as the temperature of the bulk or interface, and  (*i* = 1, 2, Γ) is a mixture-energy based equivalent. By virtue of the additive decomposition *e*_th,*i*_ + *e*_*m*,*i*_(*c*_*i*_) of the internal energy, the above definition of θ_*i*_ is consistent with the conventional definition of thermodynamic temperature as the derivative of the entropy to the internal energy.

We note that substitution of Equation 38 into Equation 37 can be recognized as a Clausius–Duhem-type inequality for the present modeling framework.

Considering next the left-hand side of Equation 37, it follows from the bulk and interface Reynolds’ transport theorems and the chain-rule of differentiation that
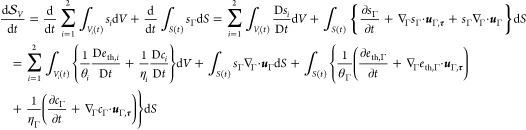
39This form now permits substitution of the balance laws for surfactant concentration and thermal energy in the bulk and on the interface, i.e., Equations 8, 9, 19, and 20
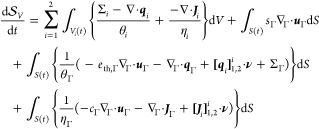
40Performing integration-by-parts on the terms involving the fluxes results in
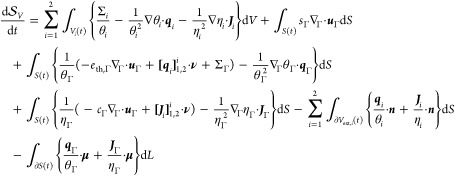
41The last two integrals now match the transport terms from Equation 38.

Substituting Equations 41 and 38 into the second law equation (37) while also substituting the expressions for the bulk and interface heat sources, Σ_*i*_ and Σ_Γ_ according to Equations 34 and 35, leads to the inequality
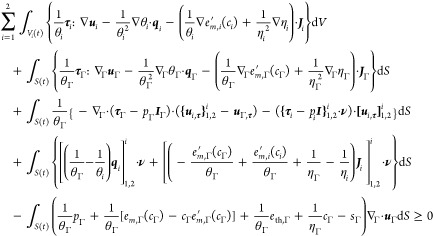
42This describes the total entropy production within the control volume at any moment during the evolution of the system. As this inequality must hold for any arbitrary control volume, the inequality must be satisfied by the bulk and interface integrands individually.

### Response Functions

In Conservation Laws section, the closure relations for the bulk traction jump and heat sources, according to (7) and (34), (35), respectively, have been derived directly from the postulated conservation laws. Completion of the PDE model (studied in its weak form in Appendix Weak formulation) still requires closure relations for the remaining auxiliary variables in Table 1, as well as equations of state for ***u***_Γ_ and *p*_Γ_, and interface conditions.

Admissible response functions are those that ensure satisfaction of the second law of thermodynamics according to Equation 42. In principle, any set of response functions that collectively guarantee the inequality in Equation 42 is admissible. Yet, more insight in the structure of the underlying entropy production mechanisms can be attained by postulating a limited parametric dependence of the remaining response functions. We therefore proceed by introducing the following minimal *constitutive class* of parametric dependences of the remaining response functions on the state variables
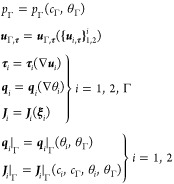
43where θ_*i*_ = θ_*i*_(*e*_th,*i*_ , *c*_*i*_) and, for notational simplicity and to elucidate the dependence of the constitutive class, we introduce the auxiliary variables
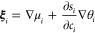
44
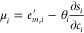
45with μ_*i*_ known as the chemical potential. In the above, the gradient operator corresponds to the surface gradient in the case of *i* = Γ.

Let us note that  (respectively ) corresponds to the trace of the heat (respectively surfactant) flux on Γ and, accordingly, the last two equations in (43) pertain to closure relations for the interface conditions. The constitutive class (43) is minimal in the sense that under further reduction of the dependences, inequality (42) is not generally satisfied or only satisfied for trivial (i.e., vanishing) response functions. In the subsequent subsections, we derive explicit expressions for the response functions in (43) subject to (42).

#### Equations of State

Consider a system state of uniform and equaling velocity and temperature fields over all domains (∇***u***_*i*_ = **0**,  = **0**, ∇θ_*i*_ = **0**, and  for *i* = 1, 2, Γ), uniform surfactant concentrations (∇*e*_*m*,*i*_^′^(*c*_*i*_) = **0** for *i* = 1, 2, Γ), and the bulk surfactant concentrations that relate to the interface concentration such that they satisfy  (see Remark 1). For such a system state, Equation 42 reduces to
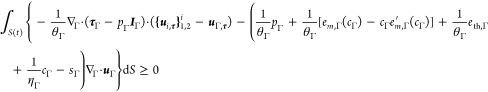
46which must still be satisfied for arbitrary instances of the state variables , θ_Γ_ and *c*_Γ_. Given that the pressure jump over the interface ∇_Γ_ · (**τ**_Γ_ – *p*_Γ_***I***_Γ_) and the interface expansion or contraction ∇_Γ_ · ***u***_Γ_ can have arbitrary signs, and the postulated dependence of ***u***_Γ,**τ**_ exclusively on , and of *p*_Γ_ exclusively on θ_Γ_ and *c*_Γ_, this can only hold generally if the following equations of state apply
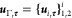
47
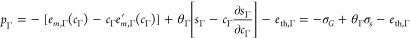
48whereby the entropy production of (46) equals zero. Let us note that the equations of state (47) and (48) are consequences of (42) in the constitutive class (43). For instance, if a different constitutive class containing the response function *p̃*_Γ_ = *p̃*_Γ_(*c*_Γ_, θ_Γ_, ∇_Γ_ ·***u***_Γ_) is considered, then Equation 48 could include a compressibility-based constitutive (dissipative) response according to *p̃*_Γ_ = *p*_Γ_ – *K*∇_Γ_ · ***u***_Γ_.

**Remark 1.** On account of state-function assumptions SA1 and SA2 in Postulates and Model Assumptions section, the derivatives *e*_*m*,*i*_^′^ are strictly increasing and η_*i*_^–1^ are strictly decreasing with respect to *c*_*i*_. Assumption SA2 moreover implies that θ_*i*_ is strictly positive. Conservation of surfactants then implies that at each interface temperature θ_Γ_, there is a unique combination of the bulk concentrations at the interface *c*_*i*_|_Γ_ and the interface concentration *c*_Γ_ such that *e*_*m*,*i*_^′^(*c*_*i*_) – θ_Γ_/η_*i*_ = *e*_m,Γ_^′^(*c*_Γ_) – θ_Γ_/η_Γ_ for *i* = 1, 2 holds.

**Remark 2.** The first term in the surface pressure (48) may be recognized as the Legendre transform of the interface energy (denoted as σ*_G_*) and the second term as the Legendre transform of the mixture entropy (as σ*_s_*). Combining Equations 48 and 21 results in the Young–Laplace pressure jump across the interface and the Marangoni effect along the interface. Specifically, (48) exhibits three contributions to these normal and tangential traction jumps, viz. a surface mixture energy effect σ*_G_*, an entropic effect θ_Γ_σ_*s*_, and a thermal effect – *e*_th,Γ_.

#### Constitutive Relations

Within the confines of the constitutive class (43), and with the equations of state for ***u***_Γ,**τ**_ and *p*_Γ_ per (47) and (48), the entropy-production inequality (42) can only be satisfied if the individual terms are non-negative, i.e.
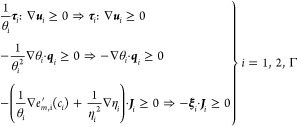
49where the gradient operator should again be interpreted as the surface gradient operator in the case *i* = Γ and **ξ** is defined in Equation 44. The implication in (49) follows from the non-negativity of the temperature, and from the convexity of *e*_*m*,*i*_ according to SA1 and the concavity of *s*_*i*_ according to SA2.

Arguably the simplest cases of constitutive relations in accordance with (49) are the linear relations. For the deviatoric stress tensor, the linear relation represents a Newtonian fluid, defined as . The inequality then holds for all symmetric positive semidefinite fourth-order tensors . The simplest example is  with μ_*i*_ > 0 as the dynamic viscosity, which leads to **τ**_*i*_ = μ_*i*_((∇***u***_*i*_) + (∇***u***_*i*_)^*T*^). If, on the interface, a compressibility-based dissipative response is added, then this is known as the Boussinesq–Scriven model. For the heat flux the linear relation is called Fourier’s law, defined as  where the inequality holds for all symmetric positive semidefinite second-order thermal conductivity tensors  the simplest example being  with λ_*i*_ > 0. The dependence of θ_*i*_ on (*e*_th,i_ , *c*_*i*_) implies that the heat flux according to Fourier’s law also includes an effect of the concentration gradient. This dependence is referred to as Dufour’s effect.^30^ The surfactant-concentration flux comprises two terms: . This reduces to Fick’s law, , in the isothermal case. In the nonisothermal case, the temperature-gradient driven flux of the species concentration is known as Soret’s effect.^30^ Again, positivity is guaranteed for positive semidefinite  , such as  with *D*_*i*_ > 0.

#### Interface Conditions

The interface conditions are derived analogously to the constitutive relations. Within the confines of the constitutive class (43), Equation 42 insists that the bulk fluxes at the interface comply with the following inequalities
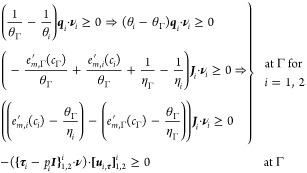
50with **ν**_*i*_ the unit normal vector on the interface external to Ω_*i*_(*t*), i.e., **ν**_1_ = **ν** and **ν**_2_ = −**ν**. The implications in the first and second line follow from multiplication with (non-negative) θ_*i*_θ_Γ_ and θ_Γ_, respectively.

The simplest interface conditions satisfying the first and third inequalities in (50) are again linear scaling relations. For the jump in the heat transfer, this is called Newton’s law of cooling:  · **ν**_*i*_ = α_*i*_ (θ_*i*_ – θ_Γ_) (*i* = 1, 2) with α_*i*_ as the heat transfer coefficient. For the traction jump, this represents the Navier slip condition:  with β_*s*_ ≥ 0 as the slip coefficient; see Remark 3 for further elaboration. The somewhat more convoluted form of the second inequality precludes a simple linear scaling relation. Instead, the simplest closure relation is
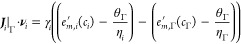
51with γ_*i*_ as the adsorption–desorption coefficient. However, in isothermal conditions the interface condition (51) admits a convenient interpretation via an equilibrium of the chemical potentials of the bulk and the interface; see Remark 4 below.

**Remark 3.** The Navier-slip condition at the interface associates an entropy production  to the deviation between the tangential velocities of the bulk components at the interface. In conjunction with the equation of state for the tangential interface velocity  in (47), this entropy-production term can be recast in an alternate form that depends on the deviation between the tangential bulk velocities at the interface and the tangential interface velocity
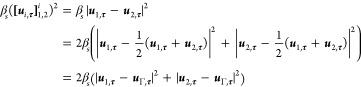
52

## Equilibrium

The characterization of equilibrium states of evolution equations with a conservation-dissipation structure, such as the presented binary-fluid-surfactant system, forms an integral part of their analysis. In this section, we investigate the implications of the derived closure relations in this regard. Additionally, we investigate concentration distribution and surface tension in equilibrium for an example system.

### Equivalences

We regard the binary-fluid-surfactant system, equipped with the equations of state (47) and (48), and the constitutive relations and interface conditions introduced in Constitutive Relations and Interface Conditions section. To characterize equilibrium solutions of the binary-fluid-surfactant system, we define three conditions that indentify an equilibrium state, and we establish that these conditions are equivalent. The equilibrium conditions are (i) entropy production vanishes uniformly in time up to exogenous terms; (ii) the system state *y* = (***u***, *p*, *c*, *e*_th_, **χ**) belongs to a certain class of equilibrium states ; (iii) the time rate of change of the state variables vanishes
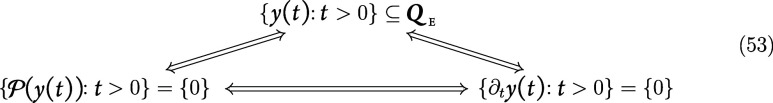
53Specifically, the first condition implies that the left member of (42), extended to all of Ω, i.e., with *V*_*i*_ = Ω_*i*_ and *S*_Γ_ = Γ, vanishes uniformly in time. The second condition connotes that equilibrium states assume a particular form, to be specified below. The third condition implies that the set of equilibrium states ***Q*** _e_ is invariant under the dynamics of the system.

**Figure 2:**
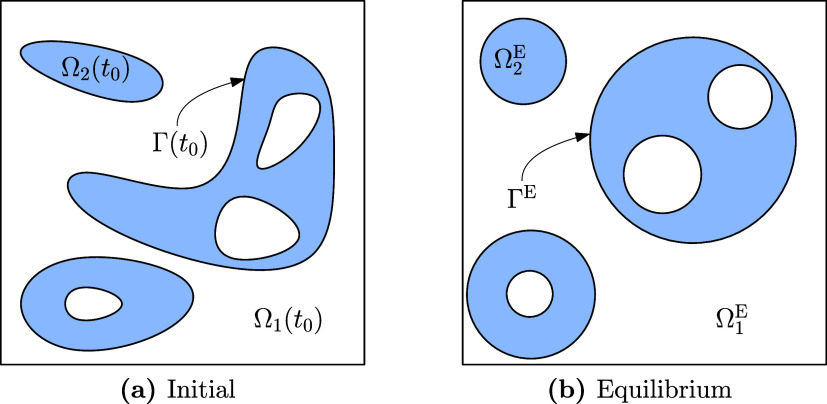
Schematic depiction of the initial and equilibrium topologies for nested spheres.

The equilibrium configuration of the binary-fluid-surfactant system depends on its initial configuration. The standing assumption that the topology of the interface does not change during the evolution of the binary-fluid-surfactant system imposes restrictions on the initial configurations and initial conditions that can be considered. For instance, it is well-known that elongated fluid filaments in 3D can exhibit instabilities which cause these filaments to break up into smaller parts; see e.g.,^46^ We, therefore, insist that the initial configuration of the interface corresponds to a collection of disconnected topological spheres such that this topological configuration is invariant under the evolution of the binary-fluid-surfactant system, i.e., for all *t* ≥ 0 it holds that Γ(*t*) = ∪_*k* = 1_^*N*_Γ_^Γ_*k*_(*t*) such that dist(Γ_*k*_(*t*), Γ_*l*_(*t*)) > 0 for *k* ≠ *l* and each Γ_*k*_(*t*) corresponds to a topological sphere; see Figure 2 for an illustration. This condition implies that the interface does not intersect the boundary ∂Ω. Without loss of generality, we assume that Ω_2_ is immersed in Ω_1_ in the sense that ∂Ω = ∂Ω_1_/Γ. Each domain Ω_*i*_(*t*) (*i* = 1, 2) is generally disconnected and comprised of *N*_*i*_ connected subsets Ω_*i*_^*k*^(*t*) = **χ**(*t*, Ω̃_*i*_^*k*^).

To specify the class of equilibrium states, we assume that the binary-fluid-surfactant system is closed by a stationary, impermeable, thermally insulated, solid boundary
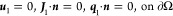
54Because (54) specifies a Dirichlet condition on the velocity on the entire external boundary ∂Ω, the pressure in the system is only defined up to a constant. To fix this constant, we insist that *p* vanishes on average
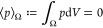
55In conjunction with these boundary conditions and under the aforementioned conditions on the configuration, we characterize the class of equilibrium states as
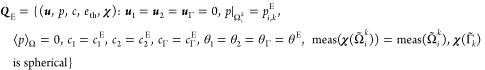
56for certain constants (·)^e^, subject to
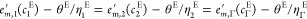
57and
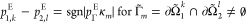
58where *p*_Γ_^e^ is the constant surface pressure at surface concentration *c*_Γ_^e^ and temperature θ^e^ according to (48), and κ_*m*_ denotes the (constant) additive curvature of the sphere **χ**(Γ̃_*m*_). By virtue of assumption SA2, the identities in (56) uniquely determine *e*_th, i_^e^ (*i* ∈ {1, 2, Γ}). The symbol sgn represents 1 (respectively −1) if **χ**(Ω̃_1_^*k*^) is interior (respectively exterior) to **χ**(Γ̃_*m*_). One may note that (57) prescribes the balance of bulk and surface concentrations as indicated in Remark 1. Equation 56 characterizes equilibrium states of the binary-fluid-surfactant system as being composed of geometrically spherical (possibly nested) droplets, immersed in an ambient fluid; the droplets are stationary, and the velocity in the binary fluid vanishes uniformly; the binary-fluid-surfactant system exhibits a homogeneous temperature; the surfactant concentration in each of the subsystems (fluids 1 and 2, and interface) is uniform, and the bulk and interface concentrations are balanced according to (57); the surface pressure is uniformly constant on the interface; the pressure is constant in each subdomain Ω_*i*_^*k*^ and the pressures in adjacent subdomains are related by the Young–Laplace condition.

Denoting by  the entropy production in the left-hand side of (42), extended to all of Ω, we first consider the implication . It is to be noted that this implication is supposed to hold subject to the evolution equations for the binary-fluid-surfactant system. Introducing the equations of state (47) and (48), and the constitutive relations and interface conditions introduced in Constitutive Relations and Interface Conditions section, we obtain
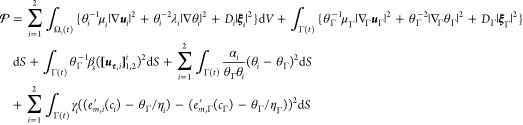
59In view of the quadratic form of the terms in (59) and the positivity of the multiplicative factors, vanishing entropy production implies
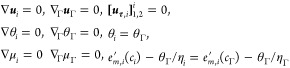
60The equalities in the left (respectively center) column of (60) imply that ***u***, θ and μ are constant in the two fluid domains (respectively on the interface). The conditions  and (17) and (47) in turn imply that ***u***_1_ = ***u***_2_ = ***u***_Γ_ = ***u***^e^. The boundary condition ***u***_1_ = 0 on ∂Ω then implies ***u***^e^ = 0. The equality θ_*i*_ = θ_Γ_ in (60) implies that the temperature is uniformly constant in Ω, i.e., θ_1_ = θ_2_ = θ_Γ_ = θ^e^. The bottom-right condition in (60) implies that the constant surfactant concentrations in the bulk domains and on the interface, *c*_1_ = *c*_1_^e^, *c*_2_ = *c*_2_^e^ and *c*_Γ_ = *c*_Γ_^e^, satisfy (57). By virtue of ***u***_*i*_ = 0 (uniformly in time), the balance of linear momentum in (7) implies the piecewise-constant form of the bulk pressures in accordance with (56). Because *c*_Γ_ and θ_Γ_ are constant, the equation of state for the surface pressure (48) implies that *p*_Γ_ is constant, and it follows from the interface momentum balance (21) that the subdomain pressures *p*_*i*,*k*_^e^ comply with the Young–Laplace relation (58). The condition ⟨*p*⟩_Ω_ = 0 that appears in (56) is intrinsically satisfied as a constraint in the system. On account of the uniformity of the subdomain pressures and the interface pressure, the interface momentum balance (21) moreover implies that the additive curvature κ is constant on each connected component of the interface, i.e., if Γ̃_*m*_ = ∂Ω̃_1_^*k*^ ∩ ∂Ω̃_2_^*l*^ ≠ 0̷ then **χ**(Γ̃_*m*_) carries a constant additive curvature, κ_*m*_. This implies that **χ**(Γ̃_*m*_) is geometrically spherical. Conservation of volume of the domain maps **χ**_*i*_ , according to ∂_*t*_ **χ**_*i*_ =  with (*t*, **χ**_*i*_^–1^(*t*, ·)) = ***u***(*t*, ·) (see Setting and Notation section) and ∇·***u***_*i*_ = 0 per (6), implies that meas( **χ**_*i*_(Ω̃_*i*_^*k*^)) = meas(Ω̃_*i*_^*k*^). Hence, we have established that .

We next consider the implication {*y*(*t*): *t* > 0} ⊆  ⇒ {∂_*t*_ *y*(*t*): *t* > 0} = {0}. This implication signifies that the class of equilibrium states is invariant under the dynamics of the binary-fluid-surfactant system, comprised of the conservation laws in the bulk domains and at the interface, equipped with the constitutive relations and equations of state. First regarding the balance laws for the bulk domains (7)–(9), one can infer that the right-hand sides of these equations vanish for states in . In combination with ***u*** = 0 in the material derivatives, it then follows that ∂_*t*_(***u***_*i*_ , *c*_*i*_ ,  *e*_th,*i*_) = 0. Moreover, from ***u***_*i*_ = 0 it immediately follows that ∂_*t*_ **χ** = 0. Considering next the balance laws for the interface (19)–(20), one infers in a similar manner as for the bulk domains that ∂_*t*_(*c*_Γ_, *e*_th,Γ_) = 0. Moreover, ∂_*t*_***u***_*i*_ = 0 in combination with (17) and the equation of state (47) implies ∂_*t*_***u***_Γ_ = 0. By virtue of ∂_*t*_(*c*_Γ_, θ_Γ_) = 0, the equation of state (48) implies ∂_*t*_*p*_Γ_ = 0. Finally, ∂_*t*_*p*_*i*_ = 0 follows from the previously derived relations in conjunction with (7) and (21); see Appendix Pressure time derivative. This corroborates the implication {*y*(*t*): *t* > 0} ⊆ ***Q*** _e_ ⇒ {∂_*t*_*y*(*t*): *t* > 0} = {0}.

Finally, we regard the implication , which completes the derivation of the equilibrium equivalences in (53). This implication is in fact a direct consequence of the second law. The evolution equations for the binary-fluid-surfactant system imply that  with  the entropy of the binary-fluid-surfactant system
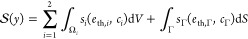
61and  the entropy production according to (59). For ∂_*t*_ *y*(*t*) = 0, the chain rule yields  and, therefore, .

### Equilibrium Example

To illustrate the equilibrium behavior of the presented binary-fluid-surfactant model, we examine the temperature dependence of the surfactant distribution and the surface tension. In this example, we assume that the volume of fluid 1 is sufficiently large in relation to the area of the interface and the volume of fluid 2, such that the surfactant concentration in fluid 1 is essentially constant irrespective of the surfactant concentration in fluid 2 and on the interface. Per Equivalences section, the system being in equilibrium implies that the surfactant concentrations are uniform in each separate domain and the temperature is uniform throughout the entire system. We assume that the interface mass is sufficiently small to neglect its thermal entropy and thermal energy. For this example, we use the mixture-energy densities and configurational-entropy densities as defined in Table 2 and the parameters in Table 3. The parameters in Table 3 correspond to a water–air system with a generic surfactant, in which water (respectively air) corresponds to fluid 1 (respectively 2). It is to be noted that in this example, the fluid-surfactant mixture is ideal: the presence of surfactants does not affect the volume of the mixture in the bulk, in accordance with assumption MA1, and the mixture energy in the bulk depends linearly on the surfactant concentration, which implies that the molar mixture-energy density of the surfactant is independent of the dilution. Fluid 1 serves as the reference level for the surfactant concentration and the energy level, i.e., the molecular energy density is zero in fluid 1. The surfactant concentration in fluid 1 is varied in the range *c*_1_^e^ ∈ [0.1, 4] mol/m^3^ and the temperature is varied in the range θ^e^ ∈ [0, 100]°C.

**Table 2:** Mixture-Energy Density and Configurational-Entropy Density for Both the Interface and Bulk Phases Used in the Model Problem

**Quantity**	**Interface**
Mixture energy	*e*_m,Γ_(*c*_Γ_) = σ_0_ + *c*_Γ_ε_Γ_
Configurational entropy	
	
**Quantity**	**Bulk**
Mixture energy	*e*_m,i_(*c*_*i*_) = *c*
Configurational entropy	

**Table 3:** Parameter Values Used in the Model Problem

**State variables**	**Symbol**	**Value**
Gas constant	*R*	8.314 J/mol K
Reference surface tension	σ_0_	72.8 mN/m
Maximum surface concentration	*c*_Γ,max_	3.4 μmol/m^2^
Reference concentration	*c*_0_	2 mol/m^3^
Molecular energy density	ε_1_	0 J/mol
Molecular energy density	ε_Γ_	–5 kJ/mol

In equilibrium, the distribution of the surfactant over the bulk domains and the interface adheres to (57) with 1/η_*i*_ = ∂*s*_*i*_/∂*c*_*i*_. Inserting the expressions in Table 2 in these identities leads to
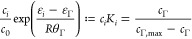
62where *K*_*i*_ = (1/*c*_0_) exp((ε_*i*_ – ε_Γ_)/*Rθ*_Γ_) is the so-called equilibrium distribution constant, or adsorption constant, which determines the surfactant distribution between the bulk and the interface in equilibrium. Noting that *R* = *k*_b_*N*_a_, with *k*_b_ as the Boltzmann constant and *N*_a_ as the Avogadro number, the ratio in the exponent in *K*_*i*_ can be conceived of as the entropy corresponding to the heat of adsorption, normalized with respect the Boltzmann constant, per mol. The equilibrium distribution constant is practically relevant in that it is a quantity that can generally be determined experimentally.^1,27^ An expression such as (62) is commonly referred to as a surfactant isotherm; see Remark 4.

**Figure 3:**
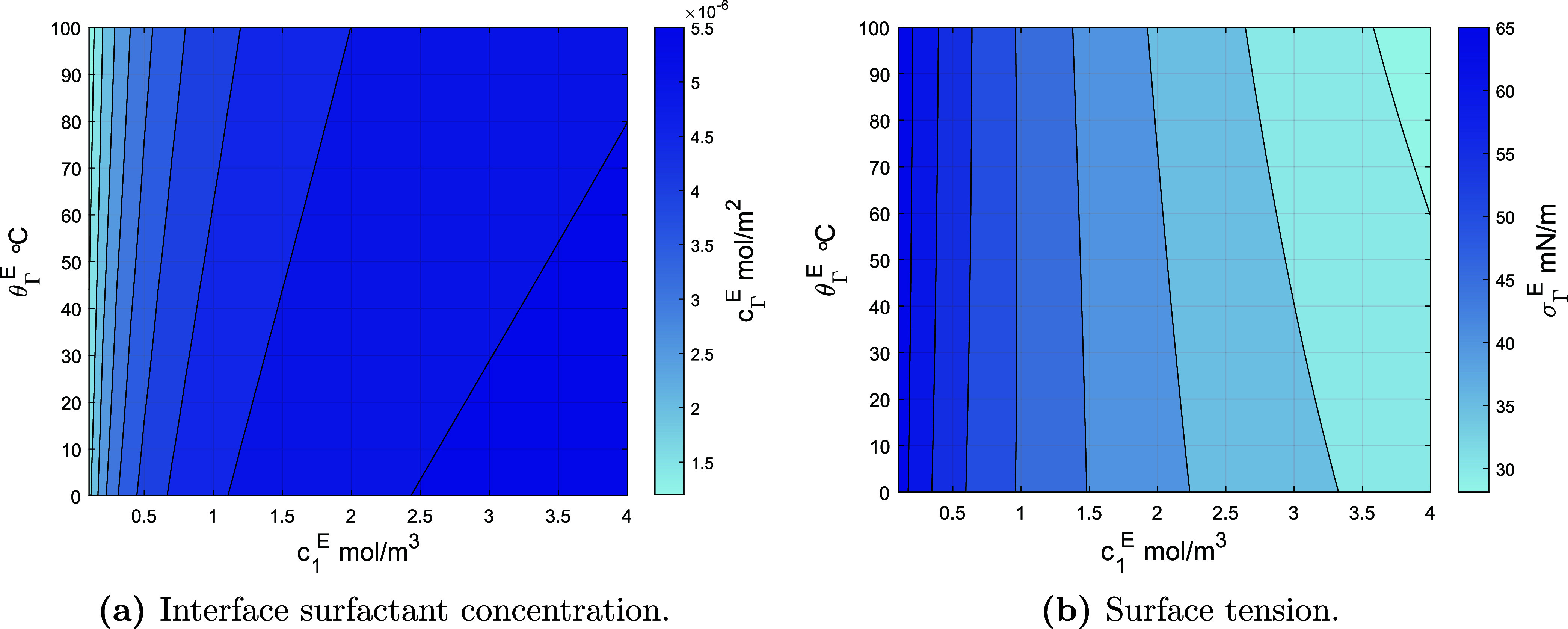
Equilibrium interface surfactant concentration and surface tension, as a function of temperature and bulk concentration of fluid 1.

Figure 3 plots the equilibrium interface surfactant concentration and the corresponding surface tension, versus the surfactant concentration in fluid 1 and the equilibrium temperature, for the models in Table 2 and the parameters in Table 3. The equilibrium concentrations follow from (62) and, in turn, the surface tension σ_Γ_ = −*p*_Γ_ follows from (48). Panel 3a conveys that at constant temperature, the interface surfactant concentration increases as the concentration in fluid 1 increases. From Panel 3b, one can observe that surface tension decreases accordingly. For higher temperatures, the equilibrium interface surfactant concentration increases slower with increasing concentration in fluid 1, due to a decreasing equilibrium adsorption factor, while the surface tension decreases faster due to the linear temperature dependency as shown in (48). One can observe that for constant surfactant concentration in fluid 1, the equilibrium surface concentration increases with increasing temperature because the equilibrium adsorption factor decreases. Panel 3b conveys that for low, constant surfactant concentration in fluid 1, the surface tension increases with increasing temperature in the considered temperature interval, while for high concentrations, the surface tension instead decreases. In the intermediate regime around *c*_1_^e^ ≈ 1 mol/m^3^, surface tension depends nonmonotonously on temperature in the considered temperature interval.

**Remark 4.** In literature, “isotherms” describe the surfactant distribution in a binary-fluid surfactant system in equilibrium.^27−29^ Many isotherms have been formulated, such as the Henry, Langmuir, Freundlich, Volmer, and Frumkin isotherms. Any particular isotherm can be more or less suitable depending on the chemical composition of the surfactant and the concentration range of the application. These isotherms are typically derived from a postulated Helmholtz free energy (*A*_*i*_ = *e*_*m*,*i*_(*c*_*i*_) – θ_*i*_*s*_*i*_), which combines energetic and entropic contributions, obtained from a partition function via statistical mechanics.^28^ The mixture energy density and configurational entropy density of Table 2 correspond directly to the well-known Langmuir isotherm. This isotherm is based on an interface with a monolayer of surfactant molecules with a maximum concentration of *c*_Γ_, max and sufficiently low bulk concentration, i.e., below the critical micelle concentration. To show this correlation, we note that under isothermal conditions, the surfactant adsorption in (51) can be expressed in terms of the chemical potentials μ_*i*_ as
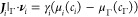
63where  corresponds to the partial derivative of the Helmholtz free-energy density to the surfactant concentration. The isotherm then represents balance of the chemical potentials: μ_*i*_(*c*_*i*_) = μ_Γ_(*c*_Γ_). Note that this is only valid in isothermal conditions, i.e., subject to θ_1_ = θ_2_ = θ_Γ_ = θ^e^.

## Conclusions

We presented a systematic continuum-thermodynamics-based derivation of a binary-fluid surfactant model, comprising two bulk fluids with an interface of codimension 1 and soluble surfactants in both fluids. We introduced the model and the underlying assumptions and postulates pertaining to the state functions. The considered model is based on the usual assumptions of vanishing mass density of the interface and constant mass density of the bulk phases. We derived the admissibility conditions on the constitutive relations of the model via a Coleman-Noll procedure. To this purpose, we specified the conservation relations for the binary-fluid-surfactant system, and the balance of entropy. We derived the constraints on the equations of state, constitutive relations and interface conditions imposed by the second law of thermodynamics, and we derived explicit forms for these response functions in the context of a minimal constitutive class. For the latter model, we regarded the characterization of equilibrium via vanishing entropy production, stationarity, and the particular form of equilibrium states, and proved equivalence between these different characterizations. Finally, we regarded an example with specific forms of the mixture energy and configurational entropy. In this example, we studied the surfactant concentrations and the surface tension in relation to temperature.

The considered model exhibits many classical aspects of the constitutive equations and interface conditions. The minimal constitutive class leads to a Newtonian-fluid relation for the deviatoric stress tensor, a Boussinesq–Scriven model for the interface stresses, Fourier’s law for the heat flux, and Fickian diffusion of the surfactants. The combination of the interface pressure and the jump of bulk stresses over the interface, results in a stress jump with a Young–Laplace pressure and a tangential Marangoni-stress contribution. The Marangoni stress contains a thermal effect and a surfactant concentration effect. The model allows for temperature jumps over the interface, and Newton’s law of cooling for the heat flux at the interface ensures thermodynamic consistency. The tangential stress at the interface and the tangential-velocity jump across the interface are connected by a Navier slip condition. Regarding the surfactant adsorption to the interface, thermodynamic consistency is ensured by imposing that the surfactant flux to the interface is proportional to the difference in the chemical potential between the bulk and the interface. The surfactant adsorption to the interface hence engenders entropic and energetic effects, and the surfactant exhibits different adsorption dynamics and different equilibrium distributions depending on the state functions of the mixture energy density and the configurational entropy. In equilibrium, the heat of adsorption, i.e., the entropy generated by the reduction of the mixture energy due to transfer of surfactant from the bulk to the interface, and the configurational entropy lost by fixation of surfactants to the interface, are balanced.

For the considered binary-fluid-surfactant model, we regarded the equivalence between three different characterizations of equilibrium, viz. vanishing entropy production, stationarity of the solution, and the structure of the solution in equilibrium. We established that equilibrium is characterized by uniformity of the temperature and vanishing of the velocity of the two fluid components and of the interface. Moreover, under suitable initial conditions, the equilibrium state is composed of geometrically spherical droplets of one fluid species immersed in the other. The surfactant concentration in each fluid domain and on the interface is constant, and the surfactant concentrations are related by balance of the chemical potentials. In addition, the pressure is piecewise constant, and complies with the Young–Laplace relation. The particular equilibrium example elucidated the influence of the equilibrium temperature on the surfactant distribution and on the surface tension.

The binary-fluid-surfactant model that we have derived in this work via the Coleman-Noll procedure, is by construction thermodynamically consistent. The model can for instance be used for simulating the dynamics of binary-fluid-surfactant systems based on a numerical implementation of the model. In future work, we will focus on extending the presented model to the diffuse-interface setting. Alternate important extensions of the model could address generalizations of the constitutive class to allow for more sophisticated behavior, or consideration of wall interactions, e.g., addressing dynamic-wetting scenarios.

## Supporting Information Available

Appendix Pressure time derivative shows how ∂_*t*_p_*i*_ = 0 in the implication {*y*(*t*): *t* > 0} ⊆ ***Q*** _e_ ⇒ {∂_*t*_*y*(*t*):*t* > 0} = {0} follows from the momentum balances (7) and (21). In Appendix Weak formulation we study the completion of the PDE model in its weak form with the implementation of closure relations for the auxiliary variables, as well as equations of state for ***u***_Γ_ and *p*_Γ_, and interface conditions (PDF).
