# Supplementary material for: Thermodynamics of Surfactant-Enriched Binary-Fluid Systems
Source: Langmuir. 2025 Jan 21;41(4):2141–55. doi: 10.1021/acs.langmuir.4c01724 (PMC11803720; doi:10.1021/acs.langmuir.4c01724)
Supplement: Supplementary file 1 — la4c01724_si_001.pdf [file la4c01724_si_001.pdf]

Supporting Information
Number of pages: 4, Number of figures, schemes, and tables: 0.

# Thermodynamics of surfactant-enriched binary-fluid systems

Tom B. van Sluijs*, Stein K.F. Stoter, E. Harald van Brummelen
*Eindhoven University of Technology, PO Box 513, 5600 MB Eindhoven, The Netherlands*

---

---

## Table of Contents

**Appendix A Pressure time derivative** **S-1**

**Appendix B Weak formulation** **S-2**

## Appendix A. Pressure time derivative

The purpose of this section is to establish the implication $q \in \boldsymbol{Q}_{\mathrm{E}} \Rightarrow \partial_t p = 0$. Considering the conservation-of-momentum equation for the bulk (7), and noting that $\rho_i$ is constant, we infer:

$$\partial_t \boldsymbol{u}_i + (\boldsymbol{u}_i \cdot \nabla)\boldsymbol{u}_i = -\frac{1}{\rho_i}\nabla p_i + \frac{1}{\rho_i}\nabla \cdot \boldsymbol{\tau}_i, \tag{A.1}$$

By substituting the constitutive relation $\boldsymbol{\tau}_i = \mu_i((\nabla \boldsymbol{u}_i) + (\nabla \boldsymbol{u}_i)^T)$ and subsequently differentiating the left and right members of equation (A.1) to $t$, one obtains:

$$\partial_t^2 \boldsymbol{u}_i + (\partial_t \boldsymbol{u}_i \cdot \nabla)\boldsymbol{u}_i + (\boldsymbol{u}_i \cdot \nabla)\partial_t \boldsymbol{u}_i = -\frac{1}{\rho_i}\nabla \partial_t p_i + \frac{\mu_i}{\rho_i}\Big(\nabla \cdot \nabla \partial_t \boldsymbol{u}_i + \nabla(\nabla \cdot \partial_t \boldsymbol{u}_i)\Big) \tag{A.2}$$

From the prior implication $q \in \boldsymbol{Q}_{\mathrm{E}} \Rightarrow \partial_t \boldsymbol{u} = 0$ (see Section Equivalences) it then follows that:

$$\partial_t^2 \boldsymbol{u}_i = -\frac{1}{\rho_i}\nabla \partial_t p_i. \tag{A.3}$$

and, therefore:

$$\sum_{i=1}^{2} \int_{\Omega_i(t)} \ddot{\boldsymbol{u}}_i \cdot \nabla \varphi \, \mathrm{d}V = \sum_{i=1}^{2} -\frac{1}{\rho_i} \int_{\Omega_i(t)} \nabla \partial_t p_i \cdot \nabla \varphi \, \mathrm{d}V, \qquad \forall \varphi \in C^1(\Omega) \tag{A.4}$$

*Corresponding author: T.B. van Sluijs (t.b.v.sluijs@tue.nl)

Integration-by-parts then yields the identity:

$$\int_{\Gamma(t)} \varphi\, \partial_t^2 [\![\boldsymbol{u}_i \cdot \boldsymbol{\nu}]\!]_{1,2}^i \,\mathrm{d}S + \int_{\partial\Omega} \varphi\, \partial_t^2 (\boldsymbol{u}_1 \cdot \boldsymbol{n}) \,\mathrm{d}S - \sum_{i=1}^{2} \int_{\Omega_i(t)} \varphi\, \partial_t^2 (\nabla \cdot \boldsymbol{u}_i) \,\mathrm{d}V$$
$$= \sum_{i=1}^{2} -\frac{1}{\rho_i} \int_{\Omega_i(t)} \nabla \partial_t p_i \cdot \nabla \varphi \,\mathrm{d}V \qquad \forall \varphi \in C^1(\Omega). \quad \text{(A.5)}$$

Because $[\![\boldsymbol{u}_i \cdot \boldsymbol{\nu}]\!]_{1,2}^i = 0$ on account of (17), $\boldsymbol{u}_1|_{\partial\Omega} = 0$ due to (54), and $\nabla \cdot \boldsymbol{u}_i = 0$ owing to (6), uniformly in time, the left-hand side of (A.5) vanishes.

We next consider the constitutive relation (21). By inserting the constitutive relations for the viscous-stress tensors in the bulk and on the interface, and subsequently differentiating the left and right members to $t$, one obtains:

$$[\![\mu_i\big((\nabla \partial_t \boldsymbol{u}_i) + (\nabla \partial_t \boldsymbol{u}_i)^T\big)]\!]_{1,2}^i \cdot \boldsymbol{\nu} - [\![p_i \boldsymbol{\nu}]\!]_{1,2}^i$$
$$= \nabla_\Gamma \cdot \mu_\Gamma \big((\nabla_\Gamma \partial_t \boldsymbol{u}_\Gamma) + (\nabla_\Gamma \partial_t \boldsymbol{u}_\Gamma)^T\big) - \nabla_\Gamma \partial_t p_\Gamma - \partial_t (p_\Gamma \kappa \boldsymbol{\nu})\,. \quad \text{(A.6)}$$

The results in Section Equivalences convey that for $q \in \boldsymbol{Q}_{\mathrm{E}}$, it holds that $\partial_t \boldsymbol{u}_i = 0$, $\partial_t \boldsymbol{u}_\Gamma = 0$, $\partial_t p_\Gamma = 0$ and $\partial_t(\kappa \boldsymbol{\nu}) = 0$. The latter is a consequence of $\partial_t \boldsymbol{\chi}_i = 0$. Equation (A.6) therefore reduces to $[\![\partial_t p_i]\!]_{1,2}^i = 0$, which implies that $\partial_t p_i$ is continuous across the interface. Under mild assumptions on the smoothness of $p_i$ in the subdomains, $\partial_t p_i$ can be conceived of as the restriction of a function $\partial_t p \in H^1(\Omega)$ to $\Omega_i$. The right-member of (A.5) then extends by continuity to $\varphi \in H^1$ and, recalling that the left member of (A.5) vanishes, one obtains:

$$\partial_t p \in H^1(\Omega): \qquad \int_{\Omega} \nabla \partial_t p \cdot \nabla \varphi \,\mathrm{d}V = 0 \qquad \forall \varphi \in H^1(\Omega)\,. \quad \text{(A.7)}$$

From the condition $\langle p \rangle_\Omega = 0$ associated with the boundary conditions (54), it moreover follows that $\langle \partial_t p \rangle_\Omega = 0$ which, in conjunction with (A.7), implies $\partial_t p = 0$.

## Appendix B. Weak formulation

Deriving the weak formulation of a partial differential equation serves as a check for the completeness of the system description: if unbounded terms remain, some conditions are unused, or the number of unknowns is not equal to the number of test functions, then this is cause for concern. In this appendix, we derive the weak formulation for the case of the linear closure relations discussed in Sections Constitutive relations, Equations of state, and Interface conditions. For ease of presentation, homogeneous boundary conditions are assumed along the exterior boundary.

By multiplying the conservation laws (7), (6), (8), (9), (19), and (20) by testfunctions, integrating over their respective domains and integrating by parts we obtain:

$$
\text{For } i \in \{1,2,\Gamma\},\ \text{find } (\boldsymbol{u}_i, p_i, c_i, e_{\mathrm{th},i}) \in \mathcal{V}_i \text{ such that } \forall\, (\boldsymbol{v}_i, q_i, w_i, \xi_i) \in \mathcal{V}_{i,0} :
$$

$$
\begin{cases}
\displaystyle\sum_{i=1}^{2} \int_{\Omega_i} \Big\{ \frac{\partial}{\partial t}(\rho_i \boldsymbol{u}_i) \cdot \boldsymbol{v}_i + \nabla \cdot (\rho_i \boldsymbol{u}_i \otimes \boldsymbol{u}_i) \cdot \boldsymbol{v}_i + \boldsymbol{\tau}_i : \nabla^s \boldsymbol{v}_i - p_i \nabla \cdot \boldsymbol{v}_i \Big\} \,\mathrm{d}V + \\
\qquad \displaystyle\int_{\Gamma} \Big\{ - \big( [\![ \boldsymbol{\tau}_i - p_i \boldsymbol{I} ]\!]_{1,2}^{i} \cdot \boldsymbol{\nu} \big) \cdot \big( \boldsymbol{v}_{\boldsymbol{\nu}} + \{\!\{ \boldsymbol{v}_{i,\boldsymbol{\tau}} \}\!\}_{1,2}^{i} \big) - \big( \{\!\{ \boldsymbol{\tau}_i - p_i \boldsymbol{I} \}\!\}_{1,2}^{i} \cdot \boldsymbol{\nu} \big) \cdot [\![ \boldsymbol{v}_{i,\boldsymbol{\tau}} ]\!]_{1,2}^{i} \Big\} \,\mathrm{d}S = 0\,, \\
\displaystyle\sum_{i=1}^{2} \int_{\Omega_i} q_i \nabla \cdot \boldsymbol{u}_i \,\mathrm{d}V = 0\,, \\
\displaystyle\sum_{i=1}^{2} \int_{\Omega_i} \Big\{ \frac{\partial}{\partial t} c_i\, w_i + \boldsymbol{u}_i \cdot \nabla c_i\, w_i - \boldsymbol{J}_i \cdot \nabla w_i \Big\} \,\mathrm{d}V + \int_{\Gamma} [\![ \boldsymbol{J}_i \cdot \boldsymbol{\nu}\, w_i ]\!]_{1,2}^{i} \,\mathrm{d}S = 0\,, \\
\displaystyle\sum_{i=1}^{2} \int_{\Omega_i} \Big\{ \frac{\partial}{\partial t} e_{\mathrm{th},i}\, \xi_i + \boldsymbol{u}_i \cdot \nabla\, e_{\mathrm{th},i}\, \xi_i - \boldsymbol{q}_i \cdot \nabla \xi_i - \Sigma_i\, \xi_i \Big\} \,\mathrm{d}V + \int_{\Gamma} [\![ \boldsymbol{q}_i \cdot \boldsymbol{\nu}\, \xi_i ]\!]_{1,2}^{i} \,\mathrm{d}S = 0\,, \\
\displaystyle\int_{\Gamma} \Big\{ \frac{\partial}{\partial t} c_\Gamma\, w_\Gamma + \nabla_\Gamma \cdot (c_\Gamma \boldsymbol{u}_{\boldsymbol{\tau}})\, w_\Gamma - \kappa\, c_\Gamma u_{\boldsymbol{\nu}}\, w_\Gamma - \boldsymbol{J}_\Gamma \cdot \nabla_\Gamma w_\Gamma - [\![ \boldsymbol{J}_i ]\!]_{1,2}^{i} \cdot \boldsymbol{\nu}\, w_\Gamma \Big\} \,\mathrm{d}S = 0\,, \\
\displaystyle\int_{\Gamma} \Big\{ \frac{\partial}{\partial t} e_{\mathrm{th},\Gamma}\, \xi_\Gamma + \nabla_\Gamma \cdot (e_{\mathrm{th},\Gamma} \boldsymbol{u}_{\boldsymbol{\tau}})\, \xi_\Gamma - \kappa\, e_{\mathrm{th},\Gamma} u_{\boldsymbol{\nu}}\, \xi_\Gamma - \boldsymbol{q}_\Gamma \cdot \nabla_\Gamma \xi_\Gamma - [\![ \boldsymbol{q}_i ]\!]_{1,2}^{i} \cdot \boldsymbol{\nu}\, \xi_\Gamma - \Sigma_\Gamma\, \xi_\Gamma \Big\} \,\mathrm{d}S = 0\,, \\
\displaystyle\int_{\Gamma} \big( \boldsymbol{u}_{\Gamma,\boldsymbol{\tau}} - \{\!\{ \boldsymbol{u}_{i,\boldsymbol{\tau}} \}\!\}_{1,2}^{i} \big) \cdot \boldsymbol{v}_\Gamma \,\mathrm{d}S = 0\,, \\
\displaystyle\int_{\Gamma} \big( p_\Gamma + \sigma_G - \theta_\Gamma \sigma_s + e_{\mathrm{th},\Gamma} \big)\, q_\Gamma \,\mathrm{d}S = 0\,.
\end{cases}
\tag{B.1}
$$

where the testfunction $\boldsymbol{v}$ is assumed to comply with the interface mass-balance relation $[\![ \boldsymbol{v}_{i,\boldsymbol{\nu}} ]\!]_{1,2}^{i} = \boldsymbol{0}$ from Equation (17).

When we consider a Newtonian fluid with Fourier and Fick's law for the heat and concentration flux, with the equations of state and interface conditions as described in Section Constitutive relations and equations of state, all the unknown and unbounded terms

can be treated by substitution of the appropriate closure relations:

For $i \in \{1,2,\Gamma\}$, find $(\boldsymbol{u}_i, p_i, c_i, e_{\text{th},i}) \in \mathcal{V}_i$ such that $\forall\, (\boldsymbol{v}_i, q_i, w_i, \xi_i) \in \mathcal{V}_{i,0}$ :

$$\begin{cases}
\sum_{i=1}^{2} \int\limits_{\Omega_i} \left\{ \frac{\partial}{\partial t}(\rho_i \boldsymbol{u}_i) \cdot \boldsymbol{v}_i + \nabla \cdot (\rho_i \boldsymbol{u}_i \otimes \boldsymbol{u}_i) \cdot \boldsymbol{v}_i + 2\mu_i \nabla^s \boldsymbol{u}_i : \nabla^s \boldsymbol{v}_i - p_i \nabla \cdot \boldsymbol{v}_i \right\} \mathrm{d}V + \\
\qquad \int\limits_{\Gamma} \Big\{ \big( \nabla_\Gamma p_\Gamma + p_\Gamma \kappa \boldsymbol{\nu} \big) \cdot \big( \boldsymbol{v}_{\boldsymbol{\nu}} + \{\!\{ \boldsymbol{v}_{i,\boldsymbol{\tau}} \}\!\}_{1,2}^{i} \big) \\
\qquad\qquad + \beta_s \, [\![ \boldsymbol{u}_{i,\boldsymbol{\tau}} ]\!]_{1,2}^{i} \cdot [\![ \boldsymbol{v}_{i,\boldsymbol{\tau}} ]\!]_{1,2}^{i} \Big\} \mathrm{d}S = 0 \,, \\
\sum_{i=1}^{2} \int\limits_{\Omega_i} q_i \nabla \cdot \boldsymbol{u}_i \, \mathrm{d}V = 0 \,, \\
\sum_{i=1}^{2} \int\limits_{\Omega_i} \left\{ \frac{\partial}{\partial t} c_i \, w_i + \boldsymbol{u}_i \cdot \nabla c_i \, w_i + \mathbb{D}_i \cdot \boldsymbol{\xi}_i \cdot \nabla w_i \right\} \mathrm{d}V + \\
\qquad \int\limits_{\Gamma} [\![ \gamma_i \left( \tfrac{\eta_i \eta_\Gamma}{\theta_\Gamma} \big( e'_{\mathrm{m,i}}(c_i) - e'_{\mathrm{m},\Gamma}(c_\Gamma) \big) + \eta_i - \eta_\Gamma \right) w_i ]\!]_{1,2}^{i} \, \mathrm{d}S = 0 \,, \\
\sum_{i=1}^{2} \int\limits_{\Omega_i} \left\{ \frac{\partial}{\partial t} e_{\text{th},i} \, \xi_i + \boldsymbol{u}_i \cdot \nabla e_{\text{th},i} \, \xi_i + \lambda_i \nabla \theta_i \cdot \nabla \xi_i \right. \\
\qquad\qquad \left. - 2\mu_i \nabla^s \boldsymbol{u}_i : \nabla^s \boldsymbol{u}_i \, \xi_i - G''_i D_i \nabla c_i \cdot \nabla c_i \, \xi_i \right\} \mathrm{d}V + \int\limits_{\Gamma} [\![ \alpha_i (\theta_i - \theta_\Gamma) \xi_i ]\!]_{1,2}^{i} \, \mathrm{d}S = 0 \,, \\
\int\limits_{\Gamma} \left\{ \frac{\partial}{\partial t} c_\Gamma \, w_\Gamma + \nabla_\Gamma \cdot (c_\Gamma \{\!\{ \boldsymbol{u}_{i,\boldsymbol{\tau}} \}\!\}_{1,2}^{i}) \, w_\Gamma - \kappa \, c_\Gamma u_{\boldsymbol{\nu}} \, w_\Gamma + \mathbb{D}_\Gamma \cdot \boldsymbol{\xi}_\Gamma \cdot \nabla_\Gamma w_\Gamma \right. \\
\qquad \left. - [\![ \gamma_i \left( \tfrac{\eta_i \eta_\Gamma}{\theta_\Gamma} \big( e'_{\mathrm{m,i}}(c_i) - e'_{\mathrm{m},\Gamma}(c_\Gamma) \big) + \eta_i - \eta_\Gamma \right) ]\!]_{1,2}^{i} \, w_\Gamma \right\} \mathrm{d}S = 0 \,, \\
\int\limits_{\Gamma} \left\{ \frac{\partial}{\partial t} e_{\text{th},\Gamma} \, \xi_\Gamma + \theta_\Gamma \sigma_s \, \nabla_\Gamma \cdot \boldsymbol{u}_\Gamma \, \xi_\Gamma + e_{\text{th},\Gamma} u_{\boldsymbol{\nu}} \kappa \, \xi_\Gamma + \lambda_\Gamma \nabla \theta_\Gamma \cdot \nabla_\Gamma \xi_\Gamma - [\![ \alpha_i (\theta_i - \theta_\Gamma) ]\!]_{1,2}^{i} \, \xi_\Gamma \right. \\
\qquad - [\![ \gamma_i \big( e'_{\mathrm{m,i}}(c_i) - e'_{\mathrm{m},\Gamma}(c_\Gamma) \big) \left( \tfrac{\eta_i \eta_\Gamma}{\theta_\Gamma} \big( e'_{\mathrm{m,i}}(c_i) - e'_{\mathrm{m},\Gamma}(c_\Gamma) \big) + \eta_i - \eta_\Gamma \right) ]\!]_{1,2}^{i} \, \xi_\Gamma \\
\qquad \left. - 2\mu_\Gamma \nabla_\Gamma^s \boldsymbol{u}_\Gamma : \nabla_\Gamma^s \boldsymbol{u}_\Gamma \xi_\Gamma - e''_{\mathrm{m},\Gamma} D_\Gamma \nabla_\Gamma c_\Gamma \cdot \nabla c_\Gamma \, \xi_\Gamma - \beta_s \, [\![ \boldsymbol{u}_{i,\boldsymbol{\tau}} ]\!]_{1,2}^{i} \cdot [\![ \boldsymbol{u}_{i,\boldsymbol{\tau}} ]\!]_{1,2}^{i} \, \xi_\Gamma \right\} \mathrm{d}S = 0 \,, \\
\int\limits_{\Gamma} \big( \boldsymbol{u}_{\Gamma,\boldsymbol{\tau}} - \{\!\{ \boldsymbol{u}_{i,\boldsymbol{\tau}} \}\!\}_{1,2}^{i} \big) \cdot \boldsymbol{v}_\Gamma \, \mathrm{d}S = 0 \,, \\
\int\limits_{\Gamma} \big( p_\Gamma + \sigma_G - \theta_\Gamma \sigma_s + e_{\text{th},\Gamma} \big) \, q_\Gamma \, \mathrm{d}S = 0 \,.
\end{cases} \tag{B.2}$$

All aforementioned closure relationships have found their way into the weak formulation, and all the unbounded interface terms in Equation (B.1) have been treated. This points to the completeness of the description of the physical system.
